# High-Performance All-Optical Logic Operations Using Ψ-Shaped Silicon Waveguides at 1.55 μm

**DOI:** 10.3390/mi14091793

**Published:** 2023-09-19

**Authors:** Amer Kotb, Kyriakos E. Zoiros, Chunlei Guo

**Affiliations:** 1 School of Chips, XJTLU Entrepreneur College (Taicang), Xi’an Jiaotong-Liverpool University, Taicang, Suzhou 215400, China; 2 Department of Physics, Faculty of Science, University of Fayoum, Fayoum 63514, Egypt; 3 Lightwave Communications Research Group, Department of Electrical and Computer Engineering, School of Engineering, Democritus University of Thrace, 67100 Xanthi, Greece; kzoiros@ee.duth.gr; 4 The Institute of Optics, University of Rochester, Rochester, NY 14627, USA

**Keywords:** logic operations, Ψ-shaped silicon waveguide, contrast ratio

## Abstract

We simulate with FDTD solutions a complete family of basic Boolean logic operations, which includes XOR, AND, OR, NOT, NOR, NAND, and XNOR, by using compact Ψ-shaped silicon-on-silica optical waveguides that are operated at a 1.55 μm telecommunications wavelength. Four identical slots and one microring resonator, all made of silicon deposited on silica, compose the adopted waveguide. The operating principle of these logic gates is based on the constructive and destructive interferences that result from the phase differences incurred by the launched input optical beams. The performance of these logic operations is evaluated against the contrast ratio (CR) metric. The obtained results suggest that the considered functions designed with the employed waveguide can be realized all-optically with higher CRs and faster speeds than other reported designs.

## 1. Introduction

In the computer and microelectronics industry, silicon is widely employed as a semiconductor in solid-state devices. Of all the semiconductor materials, silicon wafers offer the best crystal quality and the lowest cost. A silicon-based optical waveguide is a type of structure created by depositing a thin layer of crystalline silicon over an insulating layer, which is commonly made of silica (silicon dioxide). Because of the high infrared transparency of silicon and the significant difference in refractive indices between silicon (~3.45) and silica (~1.46), silicon-on-insulator (SOI) waveguides exhibit unique optical properties [1]. The SOI platform has evolved into silicon photonics for many significant reasons. For example, silicon is widely available and compatible with cutting-edge complementary metal-oxide semiconductor technology, making it possible to create structures as thin as 10 nm at a fair price [2,3,4,5,6,7]. Extremely compact optical devices can be made possible by silicon’s high optical confinement, which enables bending waveguide radii of only a few micrometers and functional waveguide elements of just ten to a few hundred micrometers [8]. On the other hand, all-optical logic gates (AOLGs) overcome the inherent limitations of their electronic equivalents, particularly the small bandwidth and low speed of data transport, thereby allowing for better information processing [9,10]. Recently, AOLGs have been realized using a variety of waveguide designs [11,12,13,14,15,16,17,18,19,20,21,22,23,24,25,26,27,28,29,30,31,32,33,34,35,36], such as photonic crystal (PC) waveguides [11,20], silicon waveguides [21,22,23,24,25,26,27,28], gold nanowire waveguides [29], gold disk-shaped nanoparticles [30], metal slot waveguide networks [31], one-dimensional metal–insulator–metal structures [32], dielectric–metal–dielectric designs [33], dielectric-loaded waveguides [34], graphene compact microdisk resonators [35], and semiconductor optical amplifiers [36], which have been employed to realize AOLGs. Each of these reported designs has its own unique design and different features, which has led to publication. The challenge that still exists is to realize multifunctional logic operations with high performance by exploiting basic and inexpensive waveguides. The research conducted for this purpose is expected to extend the suite of waveguide-based schemes that can be employed for implementing AOLGs, make available new technological options for this purpose, and open up new perspectives on realizing more complex photonic circuits at the fundamental- and system-oriented levels that rely on these gates as the core building modules. To achieve this aim, the compact waveguide that we propose in this work is capable of executing seven logic operations simultaneously, whereas the majority of the other reported designs have used PCs to implement only one or, at most, two logic operations [11,12,13,14,15,16,17,18,19,20,21,22]. Additionally, compared to the materials (i.e., silicon and silica) that we propose to be used in our design, previously reported efforts have utilized noble metals, such as gold and silver [29,30,31,32,33,34], which are less cost-effective. Moreover, these reported schemes require intricate and exceptionally accurate microfabrication technologies. Therefore, the realization of multifunctional logic operations with high performance by exploiting basic and inexpensive waveguides remains an open issue. Following prior attempts, such as those in [11,12,13,14,15,16,17,18,19,20,21,22,23,24,25,26,27,28,29,30,31,32,33,34,35,36], in this paper we account for a complete family of fundamental AOLGs, including exclusive OR (XOR), AND, OR, NOT, NOT OR (NOR), NOT AND (NAND), and exclusive NOR (XNOR), by using Ψ-shaped silicon-on-silica optical waveguides at a 1.55 μm telecommunications wavelength. The proposed Ψ-shaped waveguide is composed of one microring resonator placed between three input slots and one output slot. The constructive and destructive interferences that result from the different phases incurred by the input optical beams govern the working principle of these logic operations. The performance of the considered logic operations is assessed against the contrast ratio (CR) by means of Lumerical finite-difference-time-domain (FDTD) solutions [37], with the convolutional perfectly matched layer serving as a boundary condition [38]. In this context, the mesh accuracy of the xyz axis is adjusted to 0.05 μm, 0.05 μm, and 0.01 μm, respectively. The obtained results indicate that when these logic functions are designed using the proposed waveguide, they can operate at 120 Gb/s and with higher CRs than other reported designs [11,16,17,18,21,25,26,30,31,32,33,34]. Consequently, AOLGs and signal processing could be accomplished with comparatively better performance to efficiently meet the current and future demands of modern photonic circuits and networks.

## 2. Ψ-Shaped Silicon Waveguide

**Figure 1:**
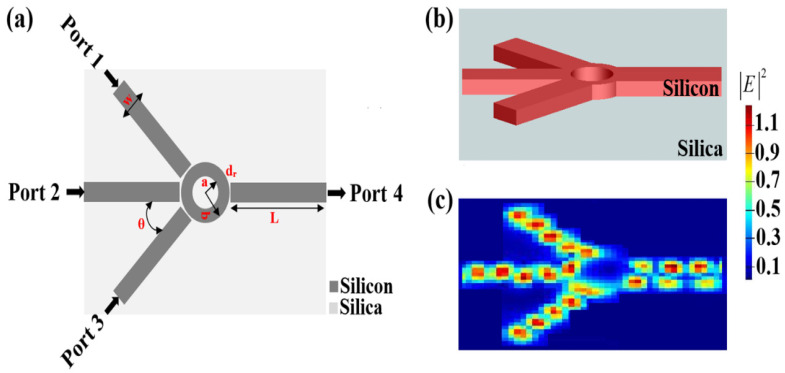
(**a**) Schematic illustration, (**b**) FDTD 3D view, and (**c**) electric field intensity distributions of the Ψ-shaped silicon waveguide.

The Ψ-shaped waveguide consists of four identical slots and one microring resonator, all made of silicon as the core patterned on a silica substrate as the cladding. On this waveguide, the microring resonator is placed in the middle between three slots as input ports and one slot as an output port. The input ports are excited by a transverse-magnetic-mode polarized wave at 1.55 μm. The input ports have the same intensity and wavelength. Inside the microring, the light that is coupled to it from the three input ports will begin to rotate. The wavelength, which is resonating inside the ring, will experience constructive interference and continue to rotate there due to the total internal reflection phenomenon. Then, this resonant wavelength can be coupled again through the output slot of port 4. The resonant wavelength (λ_s_) is given by $\lambda_{s}=4\pi n_{\mathrm{eff}}b$, where n_eff_ is the effective refractive index and b is the microring outer radius [33]. It should be noted that n_eff_ relies not only on the wavelength but also on the mode in which the light propagates. Moreover, n_eff_ clearly depends on the entire waveguide design and is not merely a material feature. Calculations in numerical mode can be used to determine the n_eff_ value [39]. The schematic illustration, FDTD 3D view, and electric field intensity distributions of the Ψ-shaped silicon waveguide are shown in Figure 1.

The electric field intensities at the input and output ports are calculated using FDTD monitors. The normalized threshold transmission (T_th_), which indicates the smallest normalized power required to create T, is set to 0.2. This value is notably higher than the values that have been used for the same purpose in other studies conducted on the treated topic [24,27,28,34]. The output spectral transmission (T) is given by $T=I_{out}/I_{in}{=\left|E_{out}\right|}^{2}/{\left|E_{in}\right|}^{2}$, where I_out_ is the intensity at the output port (i.e., port 4) and $I_{in}=I_{1}+I_{2}+I_{3}$ is the sum of the intensities at the three input ports [24,25,26,27,28]. Port 4 generates a logical output of ‘1’ only when T > T_th_ and a logical output of ‘0’ otherwise. The incident beams must satisfy phase-matching [40,41] requirements in order to maximize T. Destructive interference, on the other hand, scatters the incident beams when the phases of the latter and the waveguides are mismatched, hence producing a ‘0’ output. The CR is a critical metric for characterizing logic devices and is defined as $CR\left(dB\right)=10ln\left[P_{mean}^{1}/P_{mean}^{0}\right]$, where $P_{mean}^{1}$ and $P_{mean}^{0}$ are the mean peak powers of logic ‘1’ and ‘0’, respectively [13,14,15,16,17]. The default simulation parameters are given in Table 1 [25,26]. To ensure that these parameters used in [25,26] achieve a high CR, we have run the FDTD simulations iteratively until we are sure of their suitability.

**Figure 2:**
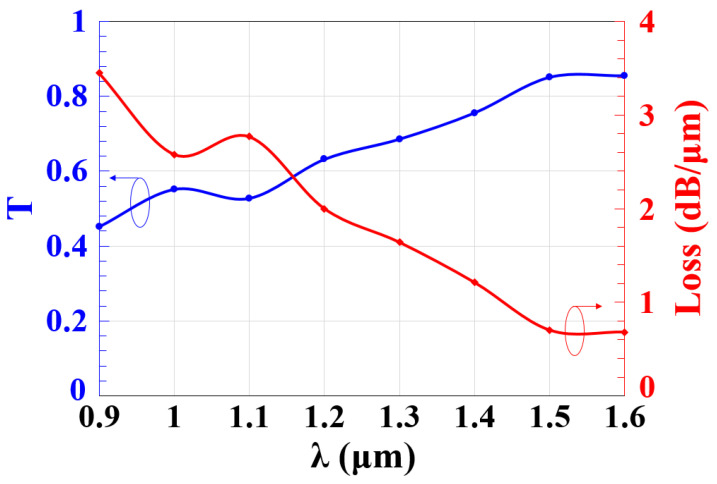
Normalized spectral transmission (T) and loss versus operating wavelength (λ) using the Ψ-shaped silicon waveguide.

The normalized spectral transmission (T) and the loss as a function of the operating wavelength (λ) are displayed in Figure 2 for the case where all the incident beams are launched at the three input ports with an identical phase of 0°. The total loss is calculated as $loss\left(dB/\mu m\right)=10log\left[1/T\right].\;$ A high T of 0.865 and a low loss of 0.63 dB/μm are achieved at 1.55 μm by employing the suggested waveguide. Such minimal propagation losses are attributed to the scattering at the slots/microring interfaces and the material absorption. The figure also shows that this waveguide achieves a high T and a low loss across the whole span of the exploitable telecommunication wavelengths, i.e., from 1.3–1.6 μm.

**Figure 3:**
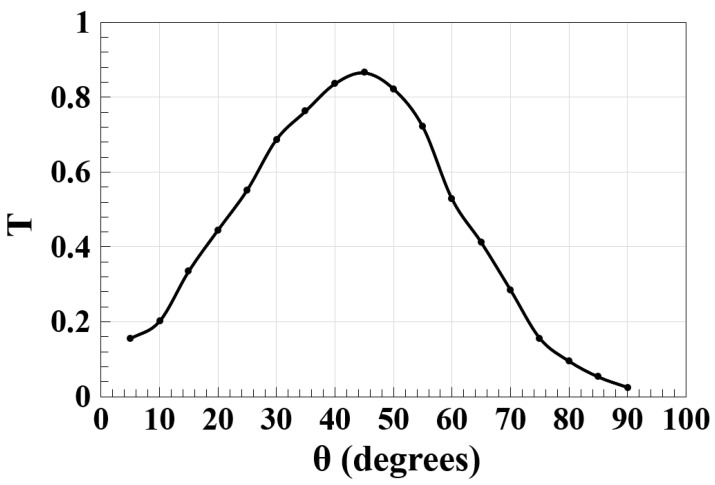
Normalized spectral transmission (T) versus angle between the slots (θ) using the Ψ-shaped silicon waveguide at 1.55 μm.

To realize the pursued logic gates with high CRs, the angle between the slots (i.e., θ) is crucial for the proposed design. As a result, Figure 3 simulates the impact of this parameter on the normalized spectral transmission (T) at a 1.55 μm operating wavelength. From this figure, it can be observed that the highest T occurs at θ = 45°, which therefore is the optimum value chosen for θ throughout our simulations. By elaborating on this figure, it can also be seen that by increasing or decreasing the value of θ, the light scattering and absorption inside the materials are increased, which in turn leads to higher losses.

**Figure 4:**
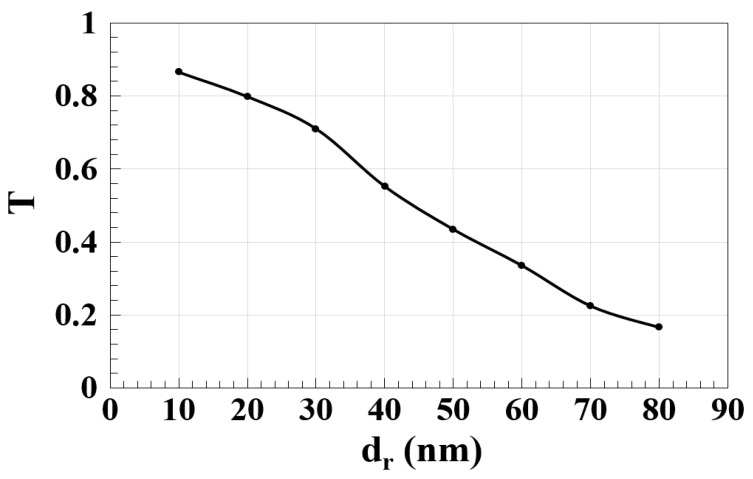
Normalized spectral transmission (T) versus distance between the slots and microring (d_r_) using the Ψ-shaped silicon waveguide at 1.55 μm.

The simulated spectral transmission (T) as a function of the distance between the slots and microring (d_r_) at 1.55 μm is shown in Figure 4. This figure shows that the Ψ-shaped silicon waveguide achieves a high T = 0.552 up to d_r_ = 40 nm. At d_r_ = 0, the proposed waveguide achieves T = 0.762, which is lower than the T = 0.865 achieved at d_r_ = 10, due to the scattering at the interfaces between the slots and the microring. This indicates that the practical realization of the proposed design should be feasible, especially with the availability of the 3D capability of the femtosecond laser direct writing (FLDW) technology [42,43,44,45,46,47,48].

**Figure 5:**
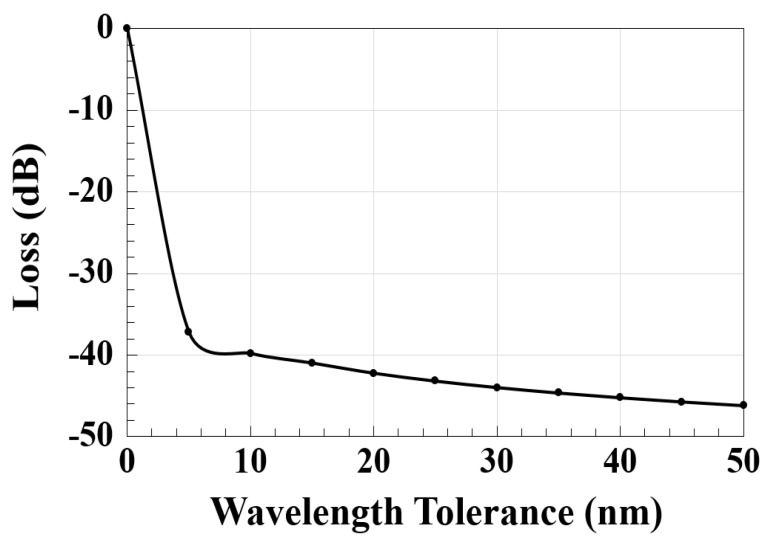
Optical loss versus wavelength tolerance using the Ψ-shaped silicon waveguide.

The manufacturing tolerances concern the control of the geometrical dimensions during processing and their impact on the functionality of the device. The operating tolerances, on the other hand, concern how the device reacts to changes in the wavelength, polarization, temperature, input field distribution, and refractive index [49,50]. The actual wavelength of a 1550 nm fiber laser may vary as 1550 nm ± 20 nm due to a wavelength tolerance of ± 20 nm [51]. The dependence of the optical loss on the wavelength tolerance using the proposed waveguide and Equations (4)–(7) from Ref. [52] is shown in Figure 5.

**Figure 6:**
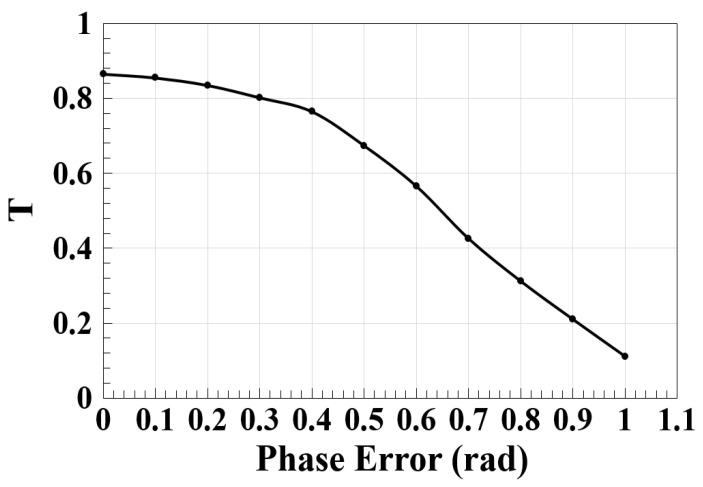
Normalized spectral transmission (T) versus phase error using the Ψ-shaped silicon waveguide at 1.55 μm.

A larger device area, apart from raising costs, also results in more insertion loss, which necessitates applying and satisfying strict requirements for the thickness and width of waveguides across the wafer in order to prevent crosstalk, which typically reduces the platforms’ performance [53]. Additionally, the variations in the process, such as those in the waveguide thickness, etching depth, waveguide width, and material refractive indices, lead to phase errors that induce uncertainty into the responses of photonic devices [54,55,56]. Therefore, it is essential to study how the phase error affects the performance of the logic operations. The dependence of the normalized spectral transmission (T) on the phase error utilizing the Ψ-shaped silicon waveguide at 1.55 μm is depicted in Figure 6. This figure demonstrates how T is decreased as the phase error is increased, which in turn leads to a reduction in the CR.

**Figure 7:**
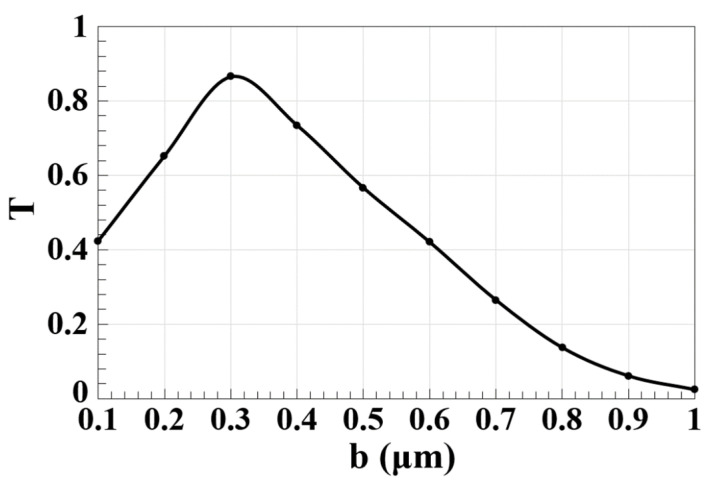
Normalized spectral transmission (T) versus microring outer radius (b) using the Ψ-shaped silicon waveguide at 1.55 μm.

We examined the design performance using various microring sizes to produce more realistic results. Therefore, in order to prevent accidental crosstalk and achieve high CRs for the considered operations, we optimized the microring outer radius (b). Figure 7 shows the dependence of T on b using the proposed waveguide at 1.55 μm. This figure clearly shows that the maximum T occurs at b = 0.3 μm, which justifies selecting this value as the optimum for b throughout the simulations. It becomes clear that as b is changed, the light scattering and materials absorption are likewise affected, which causes higher losses.

**Figure 8:**
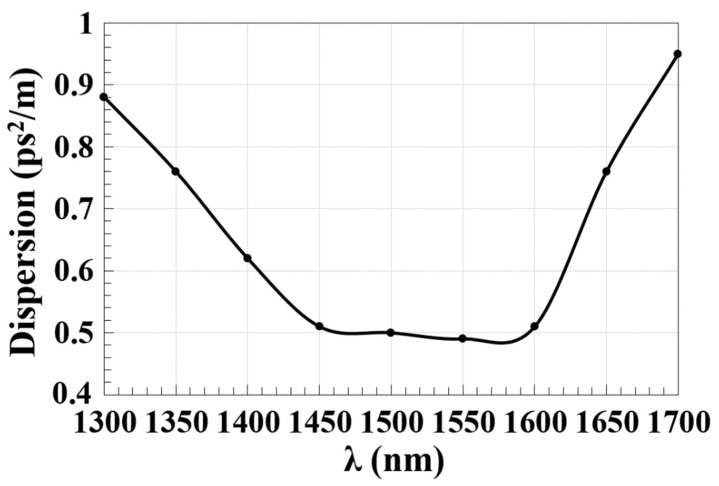
Waveguide dispersion versus operating wavelength (λ) using the Ψ-shaped silicon waveguide.

Dispersion flattening has proven to be a challenging issue to address in silicon waveguides due to the excessive waveguide dispersion and constrained light confinement in the highly nonlinear integrated waveguides. Phase mismatching can be reduced and, accordingly, the need for substantial pump power in nonlinear processes can be eliminated by improving the dispersion profile of silicon waveguides [57]. Waveguide dispersion is reduced, as seen in Figure 8, by decreasing the operating wavelength (λ). The Ψ-shaped silicon waveguide exhibits flattened dispersion from 1.45 to 1.60 μm and achieves a low dispersion of 0.49 ps^2^/m at 1.55 μm, which may be helpful for both telecom and mid-infrared applications. It is possible to control waveguide dispersion and enhance the performance of the device by modifying the waveguide’s geometry [58].

## 3. Logic Operations’ Realization

In order to perform the XOR, AND, and OR logic functions, a reference beam (REF) with a 0° must be fed into the proposed waveguide from port 2 in Figure 1. While a clock beam (Clk) with a 90° must be fed into the proposed waveguide from port 2 in Figure 1 to execute the inverted logic functions NOT, NOR, NAND, and XNOR. Either the REF (all ‘1’s) or the Clk (all ‘1’s) can be used to establish a reference phase difference between the input beams that causes either constructive or destructive interference.

### 3.1. XOR

**Figure 9:**
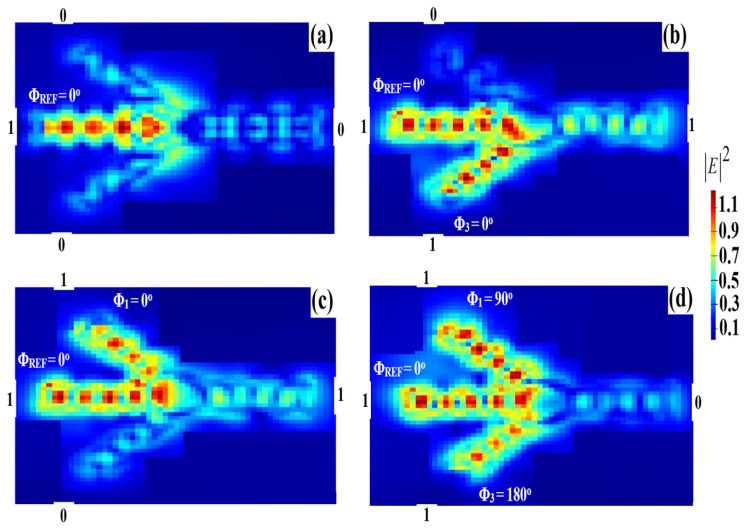
XOR field intensity distributions using the Ψ-shaped silicon waveguide at 1.55 μm: (**a**) ‘00’ input, (**b**) ‘01’ input, (**c**) ‘10’ input, and (**d**) ‘11’ input.

For the XOR operation, two input beams are injected into ports 1 and 3, respectively, while a REF is injected into port 2 in Figure 1. Constructive interference occurs when all the input beams are launched with the same phase, whereas destructive interference occurs when these beams exhibit a different phase. Therefore, when the combination (01, 10) is launched along with the REF at the same phase, i.e., Φ_1_ = Φ_3_ = Φ_REF_ = 0°, port 4 produces a ‘1’ output due to the constructive interference between the input beams. When both input beams are ‘1’, i.e., ‘11’, and are fed into the waveguide along with the REF beam at different phases, i.e., Φ_1_ = 90°, Φ_3_ = 180°, and Φ_REF_ = 0°, all the injection beams interfere destructively, resulting in T < T_th_ or equivalently in, i.e., ‘0’ output. The XOR logical outcome is achieved in this way. Figure 9 displays the XOR field intensity distributions when using the Ψ-shaped silicon waveguide at 1.55 μm.

The existence of a relative difference between $P_{mean}^{1}$ and $P_{mean}^{0}$ allows the suggested waveguide to achieve a high CR = 27 dB. The XOR simulation results obtained when utilizing the Ψ-shaped silicon waveguide at 1.55 μm are summarized in Table 2.

### 3.2. AND

**Figure 10:**
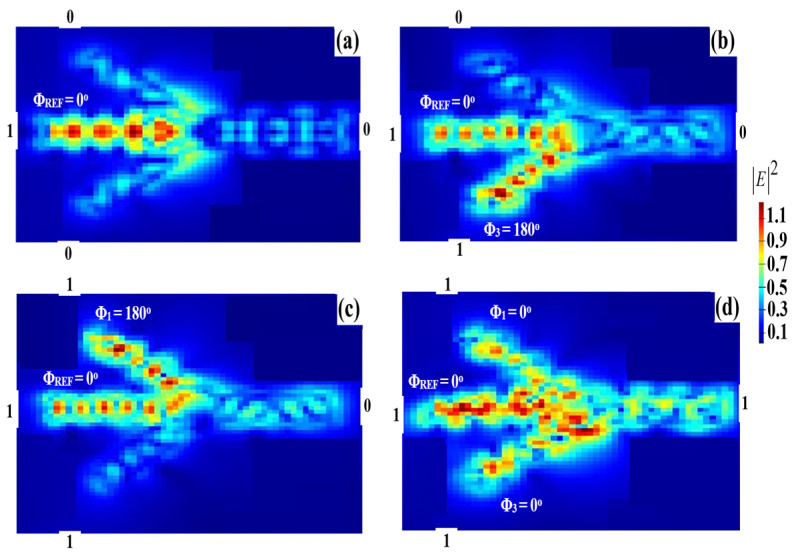
AND field intensity distributions using the Ψ-shaped silicon waveguide at 1.55 μm: (**a**) ‘00’ input, (**b**) ‘01’ input, (**c**) ‘10’ input, and (**d**) ‘11’ input.

Similar to the XOR operation, two input beams are, respectively, inserted into ports 1 and 3, as well as the REF beam (all ‘1’s) into port 2 (see Figure 1). When all the incident beams are injected at the same phase, port 4 produces a ‘1’ output due to the constructive interference between the input beams. When these incident beams are injected at a different phase, port 4 emits ‘0’ output due to the destructive interference. The AND logic operation is therefore functionally accomplished. The AND field intensity distributions when using the Ψ-shaped silicon waveguide at 1.55 μm are depicted in Figure 10.

A CR = 28.28 dB is achieved using the proposed waveguide at 1.55 μm. The rest of the AND simulation results are summarized in Table 3.

### 3.3. OR

**Figure 11:**
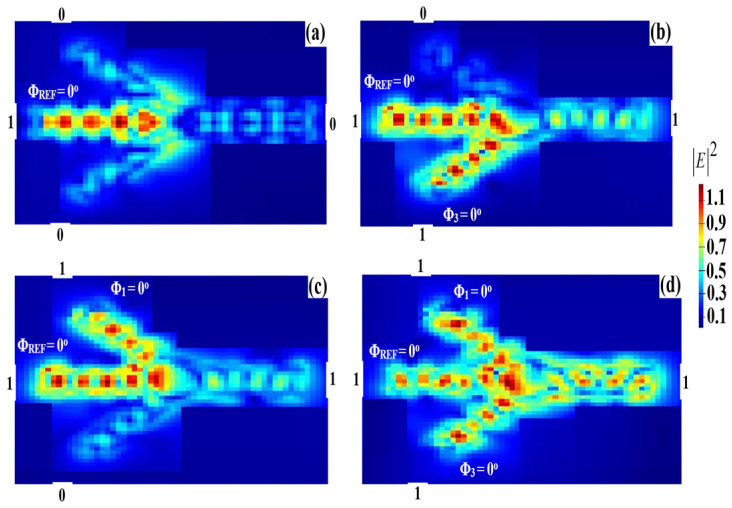
OR field intensity distributions using the Ψ-shaped silicon waveguide at 1.55 μm: (**a**) ‘00’ input, (**b**) ‘01’ input, (**c**) ‘10’ input, and (**d**) ‘11’ input.

For the OR operation, two input beams are supplied to the Ψ-shaped waveguide from ports 1 and 3, respectively, while the REF beam is supplied from port 2 in Figure 1. When the combination (01, 10, or 11) is launched with the REF beam at the same phase of 0°, the outcome becomes ‘1’ because of the constructive interference between the input beams having the same angle. Figure 11 illustrates the OR field intensity distributions when employing the proposed waveguide at 1.55 μm.

In terms of the T and CR, Table 4 displays the results of the OR simulation at 1.55 μm. The excessive difference between $P_{mean}^{1}$ and $P_{mean}^{0}$ allows us to achieve a high CR = 31 dB.

The REF beam plays an important role in achieving the XOR, AND, and OR logic operations. Table 5 displays a comparison between the results of these logic gates with and without using the REF beam (meaning port 2 has ‘0’ input) in the proposed waveguide at 1.55 μm. These results confirm that the obtained CRs are much higher with the REF beam than without it.

### 3.4. NOT

**Figure 12:**
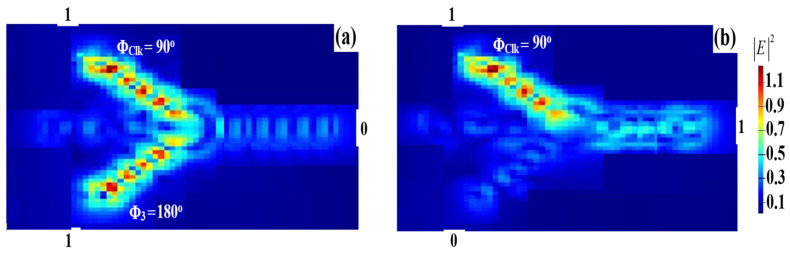
NOT field intensity distributions using the Ψ silicon waveguide at 1.55 μm: (**a**) ‘1’ input and (**b**) ‘0’ input.

To execute the NOT gate, a Clk (all ‘1’s), which comprises consecutive ‘1’s, and one beam are, respectively, fed into the Ψ-shaped waveguide from the upper and lower ports (i.e., ports 1 and 3) in Figure 1. First, the angles of the input beam and Clk should be adjusted to Φ_3_ = 180° and Φ_Clk_ = 90°, respectively. When the input beam is ‘1’, T < T_th_ occurs at port 4 because the input beams experience different phases. This results in a logical ‘0’ output. When the input beam is ‘0’, T > T_th_ occurs at port 4 because the Clk does not experience any differencing phase. This results in a logical ‘1’ output. In this manner, the NOT gate is performed. Since the Clk is fed into the structure from the upper slot (i.e., P_in1_), very high light scattering and absorption occur inside the materials, resulting in a low T = 0.285, which is the highest value achieved at the optimized Φ_Clk_ = 90°. The NOR field intensity distributions when utilizing the Ψ-shaped silicon waveguide at 1.55 μm are shown in Figure 12.

A slight difference between $P_{mean}^{1}$ and $P_{mean}^{0}$ causes a small CR = 25.17 dB. Table 6 summarizes the NOT simulation results when using the proposed waveguide at 1.55 μm. 

### 3.5. NOR

**Figure 13:**
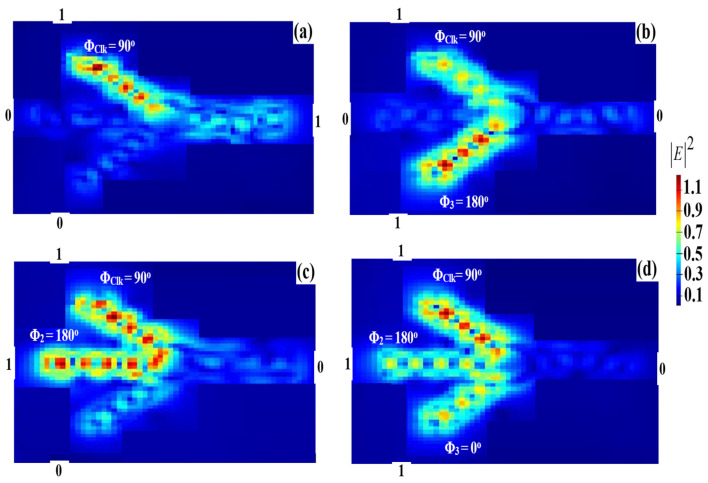
NOR field intensity distributions using the Ψ-shaped silicon waveguide at 1.55 μm: (**a**) ‘00’ input, (**b**) ‘01’ input, (**c**) ‘10’ input, and (**d**) ‘11’ input.

To execute the NOR, the Clk with Φ_Clk_ = 90° is injected into port 1, while two beams are injected into ports 2 and 3, respectively (see Figure 1). When the combination of 01, 10, or 11 is launched at different angles, the input beams interfere destructively, resulting in a logical ‘0’ as the port 4 output. When the combination of 00 is launched, the Clk will break the phase balance of the waveguide’s ports, resulting in a logical ‘1’ as the port 4 output. Thus, the Boolean NOR logic gate is implemented as shown in Figure 13.

As shown in Table 7, the proposed waveguide achieves CR = 27.24 dB for the NOR gate.

### 3.6. NAND

**Figure 14:**
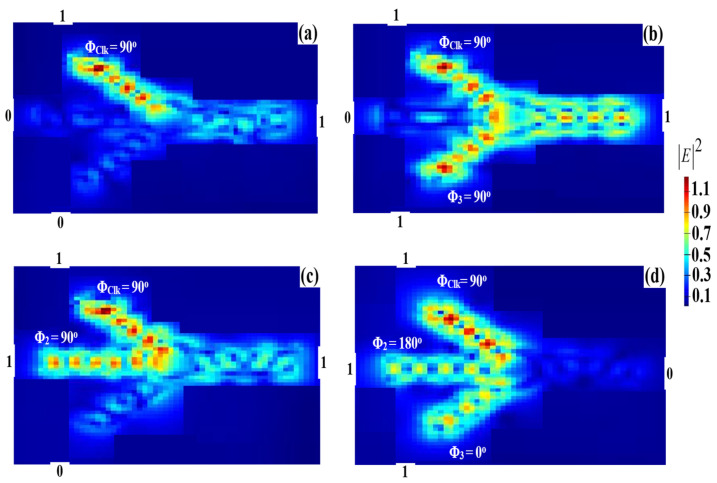
NAND field intensity distributions using the Ψ-shaped silicon waveguide at 1.55 μm: (**a**) ‘00’ input, (**b**) ‘01’ input, (**c**) ‘10’ input, and (**d**) ‘11’ input.

The NAND logic operation can be performed by injecting the Clk into port 1 and two beams into ports 2 and 3, respectively. When all the incident beams are launched at the same angle of 90°, the logic output is ‘1’ because all the input beams have the same phase and so cause constructive interference. When all the inputs have different angles, i.e., Φ_Clk_ = 90°, Φ_2_ = 180°, and Φ_3_ = 0°, destructive interference occurs between the input beams, which results in a ‘0’ output. In this way, the NAND is functionally realized at 1.55 μm, as depicted in Figure 14.

$P_{mean}^{1}$ is higher than $P_{mean}^{0}$ when using the suggested waveguide at 1.55 μm, thus leading to a high CR = 34.10 dB. Table 8 summarizes the NAND simulation results.

### 3.7. XNOR

**Figure 15:**
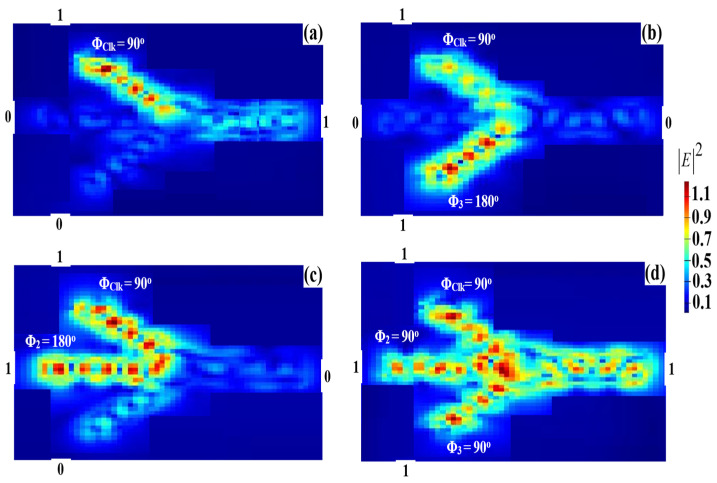
XNOR field intensity distributions using the Ψ-shaped silicon waveguide at 1.55 μm: (**a**) ‘00’ input, (**b**) ‘01’ input, (**c**) ‘10’ input, and (**d**) ‘11’ input.

For the XNOR operation, the Clk is injected to the configuration shown in Figure 1 from port 1 with Φ_Clk_ = 90°, whereas the other two beams are injected from ports 2 and 3, respectively. When the combination (11) enters together with the Clk at the same phase of 90°, port 4 produces ‘1’s as the output as a result of the concomitant constructive interference. When the combination (01) or (10) is launched with various relative phases, port 4 produces ‘0’s as the output. Figure 15 displays the XNOR field intensity distributions when using the proposed waveguide at 1.55 μm.

Owing to the significant disparity between $P_{mean}^{1}$ and $P_{mean}^{0}$, the XNOR gate has a high CR of 33.84 dB. The XNOR simulation results are listed in Table 9.

The speed of a transmission system is determined by the Nyquist formula according to $2\;B\log_{2}\left[M\right]$ [59], where M is the total number of signal levels and B is the optical bandwidth, which is specified as $B=(c/\lambda^{2})\Delta \lambda ,$ where *c* is the speed of light in a vacuum, λ = 1.55 μm is the optical carrier wavelength, and Δλ is the spectral width of the signal [25,26]. This means that the speed of the logic operations is 120 Gb/s in our case, where B = 30 GHz and M = 4 (i.e., 00, 01, 10, 11).

Owing to its mask-free, efficient, and three-dimensional capabilities, FLDW is now an established technology for photonic integrated circuit fabrication [42,43,44,45,46,47,48]. Therefore, the suggested waveguide composed of the available elements of silicon and silica could be experimentally verified in an affordable manner based on the main conclusions drawn from the conducted simulations. In fact, several AOLGs designed with a variety of waveguides have already been experimentally demonstrated [21,29,31,60,61]. Additional modules and components, such as laser sources, couplers, fibers, phase shifters, amplifiers, filters, etc., would be needed for constructing the whole logical gates’ experimental setup [62,63,64].

The ability of the suggested waveguide to realize AOLGs at various wavelengths is compared in Table 10 to that of other waveguide designs that have been reported for the same purpose. From the data cited in this table, it can be inferred that the suggested scheme may accomplish the desired logic operations with comparably higher performance and faster speed in a way that is practically feasible.

## 4. Conclusions

A set of fundamental logic functions, which include the Boolean XOR, AND, OR, NOT, NOR, NAND, and XNOR, has been designed using a Ψ-shaped silicon-on-silica waveguide and its performance has been evaluated at 1.55 μm through FDTD simulations. The conceived waveguide consists of four identical slots and one microring resonator. The constructive and destructive interferences that result from the phase differences induced by the launched input optical determine the particular logic operation and outcome. Compared to other reported waveguides, the suggested waveguide allows us to realize the specific logic functions with higher CRs and faster speeds.

## Figures and Tables

**Table 1:** Default simulation parameters [25,26].

Symbol	Definition	Value	Unit
L	Length of slot	1.0	μm
w	Width of slot	0.3	μm
d	Thickness of slot	0.3	μm
a	Microring inner radius	0.2	μm
b	Microring outer radius	0.3	μm
d_r_	Distance between slot and microring	0.01	μm
θ	Angle between slots	45	degree
λ	Operating wavelength	1.55	μm
T_th_	Threshold transmission	0.2	-

**Table 2:** XOR simulation results (T_th_ = 0.2).

Port 1	Port 3	Port 2 (REF)	T	Port 4 (Output)	CR (dB)
0	0	1	0.027	0	27
0	1	1	0.485	1	
1	0	1	0.442	1	
1	1	1	0.035	0	

**Table 3:** AND simulation results (T_th_ = 0.2).

Port 1	Port 3	Port 2 (REF)	T	Port 4 (Output)	CR (dB)
0	0	1	0.027	0	28.28
0	1	1	0.038	0	
1	0	1	0.038	0	
1	1	1	0.575	1	

**Table 4:** OR simulation results (T_th_ = 0.2).

Port 1	Port 3	Port 2 (REF)	T	Port 4 (Output)	CR (dB)
0	0	1	0.027	0	31
0	1	1	0.485	1	
1	0	1	0.442	1	
1	1	1	0.865	1	

**Table 5:** Comparison of the CR without and with the REF beam.

Operation	CR (dB) With REF	CR (dB) Without REF
XOR	27	7.2
AND	28.28	8.5
OR	31	9.7

**Table 6:** NOT simulation results (T_th_ = 0.2).

Port 1 (Clk)	Port 3	T	Port 4 (Output)	CR (dB)
1	1	0.023	0	25.17
1	0	0.285	1	

**Table 7:** NOR simulation results (T_th_ = 0.2).

Port 1 (Clk)	Port 2	Port 3	T	Port 4 (Output)	CR (dB)
1	0	0	0.285	1	27.24
1	0	1	0.019	0	
1	1	0	0.020	0	
1	1	1	0.017	0	

**Table 8:** NAND simulation results (T_th_ = 0.2).

Port 1 (Clk)	Port 2	Port 3	T	Port 4 (Output)	CR (dB)
1	0	0	0.285	1	34.10
1	0	1	0.845	1	
1	1	0	0.412	1	
1	1	1	0.017	0	

**Table 9:** XNOR simulation results (T_th_ = 0.2).

Port 1 (Clk)	Port 2	Port 3	T	Port 4 (Output)	CR (dB)
1	0	0	0.285	1	33.84
1	0	1	0.019	0	
1	1	0	0.020	0	
1	1	1	0.865	1	

**Table 10:** Comparison of the optical logic functions of the proposed and other waveguide-based waveguides.

Functions	Waveguide	Wavelength (nm)	CR (dB)	Ref.
AND, XOR, OR, NOT, NAND, NOR XNOR	PC waveguides	1550	5.42–9.59	[11]
AND, XOR, XNOR	T-shaped PC waveguides	1550	8.29–33.05	[16,17,18]
AND, NOR, XNOR	Silicon photonics platform	1550	>10 dB	[21]
XOR, AND, OR, NOT, NOR, XNOR, NAND	Silicon-on-silica waveguides	1550	20.51–30.33	[25]
XOR, AND, OR, NOT, NOR, XNOR, NAND	Silicon-on-silica waveguides	1330	11.76–32.73	[26]
XOR, NAND	Gold disk-shaped nanoparticles	451.8	24 and 26	[30]
NOT, XOR, AND, OR, NOR, NAND, XNOR	Metal slot waveguide	632.8	6–16	[31]
NOT, XOR, AND, OR, NOR, NAND, XNOR	Metal–insulator–metal structures	632.8	15	[32]
NOT, XOR, AND, OR, NOR, NAND, XNOR	Dielectric–metal–dielectric design	900 and 1330	5.37–22	[33]
XOR, AND, OR, NOR, NAND, XNOR	Dielectric-loaded waveguides	471	24.41–33.39	[34]
XOR, AND, OR, NOT, NOR, XNOR, NAND	Ψ silicon waveguides	1550	25.17–34.10	This work

## Data Availability

Not applicable.
